# Linear and non-linear analyses of Conner’s Continuous Performance Test-II discriminate adult patients with attention deficit hyperactivity disorder from patients with mood and anxiety disorders

**DOI:** 10.1186/s12888-016-0993-4

**Published:** 2016-08-11

**Authors:** Ole Bernt Fasmer, Kristin Mjeldheim, Wenche Førland, Anita L. Hansen, Vigdis Elin Giæver Syrstad, Ketil J. Oedegaard, Jan Øystein Berle

**Affiliations:** 1 Division of Psychiatry, Haukeland University Hospital, Bergen, Norway; 2 Department of Clinical Medicine, Section for Psychiatry, University of Bergen, Bergen, Norway; 3 K.G. Jebsen Centre for Research on Neuropsychiatric Disorders, Bergen, Norway; 4 Madlamarkveien 2a, Hafrsfjord, Norway; 5 Lagårdsveien 91, Stavanger, Norway; 6 Department of Clinical Psychology, University of Bergen, Bergen, Norway; 7 Centre for Research and Education in Forensic Psychiatry, Haukeland University Hospital, Bergen, Norway; 8 Department of Neuroscience, the Norwegian University of Science and Technology, Trondheim, Norway; 9 Department of Clinical Medicine, Section for Psychiatry, University of Bergen, Haukeland University Hospital, N-5021 Bergen, Norway

**Keywords:** ADHD, Cyclothymic temperament, Continuous performance test, Variability, Complexity

## Abstract

**Background:**

Attention Deficit Hyperactivity Disorder (ADHD) is a heterogeneous disorder. Therefore it is important to look for factors that can contribute to better diagnosis and classification of these patients. The aims of the study were to characterize adult psychiatric out-patients with a mixture of mood, anxiety and attentional problems using an objective neuropsychological test of attention combined with an assessment of mood instability.

**Method:**

Newly referred patients (*n* = 99; aged 18–65 years) requiring diagnostic evaluation of ADHD, mood or anxiety disorders were recruited, and were given a comprehensive diagnostic evaluation including the self-report form of the cyclothymic temperament scale and Conner’s Continuous Performance Test II (CPT-II). In addition to the traditional measures from this test we have extracted raw data and analysed time series using linear and non-linear mathematical methods.

**Results:**

Fifty patients fulfilled criteria for ADHD, while 49 did not, and were given other psychiatric diagnoses (clinical controls). When compared to the clinical controls the ADHD patients had more omission and commission errors, and higher reaction time variability. Analyses of response times showed higher values for skewness in the ADHD patients, and lower values for sample entropy and symbolic dynamics. Among the ADHD patients 59 % fulfilled criteria for a cyclothymic temperament, and this group had higher reaction time variability and lower scores on complexity than the group without this temperament.

**Conclusion:**

The CPT-II is a useful instrument in the assessment of ADHD in adult patients. Additional information from this test was obtained by analyzing response times using linear and non-linear methods, and this showed that ADHD patients with a cyclothymic temperament were different from those without this temperament.

## Background

Attention Deficit Hyperactivity Disorder (ADHD) is a heterogeneous disorder [1] and subgrouping traditionally occurs according to symptoms that follow DSM-criteria [2]. However, it is important to look for other factors that can contribute to a better understanding of the biological basis of this disorder [3]. Mood instability may be one such factor, and although it is not one of the core criteria of ADHD it is an important clinical characteristic [4] of these patients. This may represent a possible link to bipolar spectrum disorders [5], that include bipolar I, bipolar II, cyclothymia and cyclothymic temperament [6]. The cyclothymic temperament (CT) is characterized by mood instability, with intermittent cycles with rapid oscillations between elevated and depressed mood that characterize the habitual functioning of the individual, and is present since at least early adulthood [7]. This temperament, defined according to the criteria of Akiskal et al. [8, 9], is common in adult ADHD patients [6]. Positive scores on a self-evaluation form for this temperament separates a subgroup of ADHD patients with higher scores on ADHD symptom scales, a higher frequency of psychiatric symptoms, including drug and alcohol abuse, and more impairment, reflected in lower education and a reduced chance of being employed [6].

The diagnosis of ADHD is based on symptoms and behavioural criteria [2], but neuropsychological tests, such as continuous performance tests, are often employed in the assessment [10]. The Conner’s Continuous Performance Test (CPT) is one such test, however opinions differ as to how useful it is in the assessment of patients presenting for evaluation of ADHD symptoms [11–13].

The CPT uses several different measures to characterize the response patterns, one of them being variability. In neuropsychological tests ADHD patients are characterized by increased intraindividual variability of reaction times. One reason for this variability is the presence of some very long response times. In order to investigate this phenomenon further different analysis methods have been used, such as frequency domain analyses and ex-Gaussian analyses [14]. A complementary approach can be to study the complexity of times series [15, 16], employing methods from the field of non-linear systems. In general biological systems can seldom be fully characterized by using simple linear models, and other mathematical tools are usually required, obtained from the field of non-linear systems, complexity theory and chaos theory. Increased order and regularity may be a characteristic feature of diseases affecting different physiological systems [17]. On this background, non-linear methods, such as different measures of complexity and entropy, have in recent years been employed to analyze and evaluate biological time series. These methods are able to give information in addition to that obtained by traditional linear methods [15], and can be used to identify underlying mechanisms of the system being studied, and to predict how the system will change over time. For instance, in cardiology, reduced complexity, measured with approximate entropy and sample entropy, has been found to predict ventricular arrhythmias death [18]. Increased variability and changes in complexity measures have been found in patients with mood disorders using actigraph registrations [15, 19], and depressed patients have shown increased variability of reaction times in a neuropsychological test. We therefore wanted to see if we could find similar changes in ADHD patients with mood instability, defined by the presence of CT.

Thus, in this study we wanted to further characterize the time series from the CPT-II using linear and non-linear methods [15]. Reduced complexity of reaction times in a disorder of attention such as ADHD would be in line with the idea that diseases are characterized by increased order and regularity [17], and our a priori hypothesis was therefore that ADHD patients would show reduced complexity.

The aims of the study have been two-fold, first to characterize an adult population of ADHD patients according to scores on the CPT-second edition (CPT-II) [20], and to compare these patients to a clinical control group, with a mixture of mood, anxiety and attention problems.

Secondly we aimed to examine whether ADHD patients, grouped according to the presence or not of CT, differ in their profile of CPT-II scores.

## Method

### Ethics statement

The study protocol was approved by the Norwegian Regional Medical Research Ethics Committee West. Written informed consent was obtained from all participants involved in the study.

### Subjects

Patients were recruited from the private psychiatric practice of authors KM and WF, both of them certified psychiatrists with long-standing clinical experience. The patients were consecutive new referrals, between 18 and 65 years of age, in need of diagnostic evaluation of ADHD, mood or anxiety disorders.

Exclusion criteria were inability to speak Norwegian and not being able to comply with the study protocol. None were excluded because of inability to speak Norwegian. A total of 104 patients were recruited and 100 of them were tested with CPT-II. One of the patients had been treated with stimulants during testing and was therefore omitted from the analyses, bringing the total number of patients to 99, and these are reported on in the present paper. The group consisted of 48 women and 51 men, with an average age of 37.8 ± 11.0 years (mean ± SD), range 17–61.

74 % of the ADHD patients and 65 % of the clinical controls were not prescribed psychotropic medications. A summary of the drugs used by the patients is presented in Table 1.

**Table 1:** Psychotropic drug treatment (n, %)

	ADHD (*n* = 50)^a^	Clinical controls (*n* = 49)^a^
Antidepressants	10 (20 %)	10 (20 %)
Anxiolytics	3 (6 %)	2 (4 %)
Hypnotics	1 (2 %)	2 (4 %)
Antipsychotics	0 (0 %)	2 (4 %)
Lithium	0 (0 %)	2 (4 %)
Mood stabilizers except lithium	0 (0 %)	4 (8 %)
No psychotropic drug treatment	37 (74 %)	32 (65 %)

^a^ Some patients received more than one type of medication, and the total for each group therefore exceeds 100%

After a comprehensive evaluation and consensus discussion, 50 patients fulfilled criteria for a diagnosis of ADHD, while 49 did not. All these 49 patients were given other psychiatric diagnoses, (clinical controls). The gender ratio (male/female) and mean age are similar in the two groups (Table 2).

**Table 2:** Characteristics of the clinical sample

	ADHD (*n* = 50)^a^	Clinical controls (*n* = 49)^a^	P	*d*
Age (mean ± SD)	37.7 ± 10.3	37.8 ± 11.8	NS	
Gender (m/f)	28/22	23/26	NS	
WURS (mean ± SD)	52.0 ± 18.8	28.9 ± 15.3	<0.001	1.34
ASRS (mean ± SD)	50.0 ± 12.2	31.6 ± 12.7	<0.001	1.48
HADS				
Depression (mean ± SD)	4.7 ± 3.7	5 .2 ± 3.9	NS	−0.13
Anxiety (mean ± SD)	9.9 ± 4.5	9.1 ± 4.8	NS	0.17
MADRS (mean ± SD)	13.4 ± 7.2	13.9 ± 8.1	NS	0.07
MDQ + (%) ^b^	47	30	NS	
CT + (%)^c^	59	52	NS	
Bipolar disorder (%)	30	42	NS	
Unipolar depression (%)	32	29	NS	
Anxiety disorder (%)	38	60	0.026	
Alcohol or drug abuse (%)	26	13	NS	
Other diagnoses (%)	28	29	NS	

Student’s t-test or Chi square test were used to compare the two groups

^a^ The number of patients in each group varies somewhat for the different questionnaires and tests (WURS 48/45, ASRS 49/45, HADS 47/45, MADRS 49/47, CT 49/46)

^b^ Positive score on the Mood Disorder Questionnaire (MDQ)

^c^ Positive score on the scale for Cyclothymic temperament (CT)

### Psychiatric assessment

All diagnostic assessments of the patients were performed by either KM or WF using a clinical interview, supplemented when possible with information from collateral sources (relatives) concerning symptoms of ADHD in childhood. The final diagnostic evaluation assessment was made after an assessment of all available information, and a consensus diagnosis, based on DSM-IV criteria, was made after discussion of each case (KM, WF, OBF and JØB).

The following assessment instruments were employed:

- *The Mini-International Neuropsychiatric Interview (MINI Plus, version 5.0.0),* a module based semi-structured interview for DSM-IV and ICD-10 diagnoses, including a module on ADHD symptoms. All the modules except the one on psychotic disorders were administered [21, 22].
- *Wender Utah Rating Scale, 25 questions version (WURS-25),* is a 25-item self-rating scale that assesses symptoms and signs of ADHD in childhood, using a scale of 0–4 (0 = never, 4 = very often), yielding a range of scores from 0 to 100. WURS-25 has been used in previous studies in Norway [23].
- *Adult ADHD Self-Report Scale (ASRS).* This is the World Health Organization’s rating scale for current symptoms of ADHD in adults. It consists of 18 questions that follow the DSM-IV criteria for ADHD, and uses a 5 point scale of 0–4 (0 = never, 4 = very often), with a range of scores from 0 to 72. The items from 1 to 9 cover symptoms of inattention and the items from 10 to 18 cover hyperactivity and impulsivity [23–25].
- *Hospital Anxiety and Depression Scale (HADS).* This is a self-assessment form for detecting current states of depression and anxiety, and has been extensively used, also in Norway [26].
- *The Montgomery-Asberg Depression Rating Scale (MADRS),* a standard instrument for the assessment of depression [27].
- *Mood Disorder Questionnaire (MDQ).* This is a screening instrument for bipolar disorder that has been validated in both clinical and control populations. It is a self-report form with 13 questions scored “Yes” of “No”. Positive answer to at least 7 questions and confirmation that the symptoms have occurred together and caused problems is suggestive of a bipolar disorder [23, 28].
- *Cyclothymic temperament scale is* a self-report form consisting of 21 questions covering the cyclothymic temperament according to the definition of Akiskal [6, 9]. The scale is part of the larger TEMPS-A auto questionnaire [8].
- *Conner’s Continuous Performance Test II (CPT-II)* is a computerized test [20] with stimuli consisting of letters presented for 250 msec. The test comprises 360 trials, across six blocks. The time interval (1, 2, or 4 s) between the presented letter varies within each block. Subjects are instructed to press the spacebar for all letters except X, and target and nontarget letters appear randomly. The CCPT-II takes 14 min to complete. The following measures are reported: Reaction time (RT) for correct responses, numbers (%) of omission and commission errors, variability of the standard error, that is the amount of variability the individual shows in the separate segments of the test in relation to the overall standard error and a clinical ADHD Confidence Index score (range 0–100). In addition to these measures, generated by the software supplied by the producer, we have extracted raw data from the test, that is continuous series of 360 reaction times from each patient. Missing data were replaced with the mean of all the other RTs in the series. Patients with more than three successive missing responses were excluded (*n* = 11; ADHD: *n* = 8, clinical controls : *n* = 3). Since we have included information from the CPT-II test in the diagnostic assessment of ADHD this may of course have influenced the association between ADHD and CPT-II measures. We have therefore made separate correlation analyses between scores on the ADHD module of MINI + and CPT-II results.

### Mathematical analyses

For the time series raw data we calculated six measures. Linear measures: 1) The mean RT,2) the standard deviation (SD) of RT, expressed as percent of the mean RT, 3) the root mean square successive differences (RMSSD), expressed as percent of the mean RT and 4) skewness. Non-linear measures: 5) sample entropy, 6) symbolic dynamic analysis.

#### Sample entropy

For the analysis of sample entropy the data were normalized, by transforming the time series to have sample mean 0 and sample variance 1. The software used for the estimation of sample entropy was from the Physio Toolkit Research Resource for Complex Physiologic signals [29], see http://www.physionet.org. Sample entropy is a nonlinear measure, indicating the degree of regularity (complexity) of time series, and is the negative natural logarithm of an estimate of the conditional probability that subseries of a certain length (m) that match point-wise, within a tolerance (r), also match at the next point. We chose the following values, m = 2 and *r* = 0.2. Sample entropy was employed since it can be employed with comparatively short time series (>50) and is robust with regard to outliers [30].

#### Symbolic dynamics

The time series were transformed into series of symbols according to the method described by Guzzetti et al. [31] and Porta et al. [32]. The difference between the maximum and minimum value was divided into 6 equal portions (1–6), and each value of the series was assigned a number from 1 to 6, so that the transformed time series consisted of a string of numbers from 1 to 6. To reduce the problem with outliers the maximum value was set at no more than the mean +3 times the SD, and the minimum value to no less than the mean –3 times the SD. The series were then divided into overlapping sequences of three consecutive numbers. The series thus contained 358 such sequences, and the number of different sequences was counted, giving an indication of the complexity of the time series [33]. The maximum number of different sequences for such series will be 216.

### Statistics

Student’s t-test was used to evaluate differences between two groups, with the p-value set at 0.05. The Mann–Whitney U test was used when data were not normally distributed. Chi square test was employed when dealing with categorical data. Analysis of variance (ANOVA), with post-hoc Bonferroni corrections were used to compare four groups. We have not used Bonferroni corrections for the other analyses since many of the measures are correlated. Where relevant we have used ANCOVA to adjust for medication (use or no use of medication). There were no interactions with medication use for any of the measures reported. Effect sizes (d) are indicated where relevant. Pearson’s correlation coefficient was employed to evaluate correlations. SPSS version 21 was used for the statistical analyses.

## Results

### Clinical characteristics

Table 2 shows the clinical characteristics of the two groups. The ADHD group had substantially higher scores on both the WURS-25 (52.0 ± 18.8 vs. 28.9 ± 15.3, *p* < 0.001, *d* = 1.34) and ASRS scales 50.0 ± 12.2 vs. 31.6 ± 12.7, *p* < 0.001, *d* = 1.48) than the controls. For the questionnaires (HADS, MDQ, CT) and the MADRS scale there were no significant differences between the groups. The clinical diagnoses were based on the MINI+ interview. There was a higher number of patients with anxiety disorders in the group without ADHD (60 % vs. 38 %, *p* = 0.026). In the ADHD group 62 % did not have an anxiety disorder. For the other diagnoses there were no significant differences between the two groups.

#### CPT-II performance for ADHD vs. control groups

In Table 3 the results from the CPT-II test are presented. The reaction time was not different for ADHD patients compared to the clinical controls, but the values for omission (3.5 ± 5.0 vs. 0.8 ± 1.1, *p* < 0.001, *d* = 0.74) and commission errors ( 55.5 ± 24.2 vs. 35.0 ± 18.6, 9 < 0.001, *d* = 0.95) and variability (14.9 ± 14.2 vs. 6.6 ± 4.2, *p* < 0.001, *d* = 0.79) were all significantly higher in the ADHD group. The ADHD Confidence Index score was also elevated (65.0 ± 21.8 vs. 44.5 ± 20.3, *p* < 0.001, *d* = 0.97). Skewness (2.78 ± 2.52 vs. 1.91 ± 2.37, *p* = 0.011, *d* = 0.36) and the two variability measures SD (33.1 ± 11.5 vs. 24.0 ± 8.0, *p* < 0.001, *d* = 0.93), and RMSSD (40.0 ± 16.2 vs. 28.9 ± 10.8, *p* < 0.001, *d* = 0.81) showed higher values in the ADHD patients, combined with lower values for the two complexity measures sample entropy (1.57 ± 0.25 vs. 1.77 ± 0.20, *p* < 0.001, *d* = −0.89) and symbolic dynamics (76.6 ± 16.2 vs. 89.0 ± 16. 2, *p* < 0.001, d = −0.77).

**Table 3:** Patients with ADHD compared to clinical controls

I Traditional CPT-II results				
	ADHD (*n* = 50)	Clinical controls (*n* = 49)	P	*d*
Reaction time	386 ± 102	390 ± 74	NS	−0.04
Omissions (%)	3.5 ± 5.0	0.8 ± 1.1	0.001	0.74
Commisions (%)	55.5 ± 24.2	35.0 ± 18.6	< 0.001	0.95
Variability	14.9 ± 14.2	6.6 ± 4.2	< 0.001	0.79
ADHD Confidence Index	65.0 ± 21.8	44.5 ± 20.3	< 0.001	0.97
II Analyses based on extraction of raw data from the CPT-II test				
	ADHD (*n* = 42)	Clinical controls (*n* = 46)	P	*d*
Reaction time	380 ± 103	389 ± 75	NS	0.01
SD (% of mean)	33.1 ± 11.5	24.0 ± 8.0	< 0.001	0.93
RMSSD (% of mean)	40.0 ± 16.2	28.9 ± 10.8	< 0.001	0.81
Sample entropy	1.57 ± 0.25	1.77 ± 0.20	< 0.001	−0.89
Symbolic dynamics	76.6 ± 16.2	89.0 ± 16.2	0.001	−0.77
Skewness	2.78 ± 2.52	1.91 ± 2.37	0.011 ^a^	0.36

^a^ MannWhitney U-test, t-test for the other data

#### CPT-II performance for ADHD subgroups

The only significant difference between the groups two subgroups of ADHD patients, combined type and inattentive type, was in the reaction time, which was higher in the combined subtype (419 ± 120 vs. 346 ± 58, *p* = 0.008, d = 0.76). Among the patients with combined type ADHD 17 of 26 (65 %) have positive scores on the CT questionnaire compared to 12 of 23 (52 %) in the inattentive group, but this difference was not significant.

#### Correlations between CPT-II results and scores on the ADHD adult module of MINI+

Table 4 shows correlations between results from the CPT-II test and sum scores on the ADHD adult module of MINI+ . Concerning the traditional CPT-II results there were significant correlations between ADHD scores and omission errors (0.391, *p* = < 0.001), commission errors (0.395, *p* < 0.001), variability (0.330, *p* = 0.001) and ADHD Confidence Index scores (0.430, *p* < 0.001). There were significant positive correlations between ADHD scores and values for the two variability measures SD (0.315, *p* = 0.004) and RMSSD (0.284, *p* = 0.009), and significant negative correlations between ADHD scores and the two complexity measures sample entropy (−0.316, *p* = 0.004) and symbolic dynamics (−0.316, *p* = 0.004). We have not applied any post-hoc corrections to these analyses.

**Table 4:** Correlations with sum scores on the ADHD adult module of MINI+

I Traditional CPT-II results (*n* = 94)		P
Reaction time	−0.024	NS
Omissions	0.391	<0.001
Commisions	0.395	<0.001
Variability	0.330	0.001
ADHD Confidence Index	0.430	<0.001
II Analyses based on extraction of raw data from the CPT-II test (*n* = 83)		P
Reaction time	−0.110	NS
SD (% of mean)	0.315	0.004
RMSSD (% of mean)	0.284	0.009
Sample entropy	−0.316	0.004
Symbolic dynamics	−0.329	0.002
Skewness	0.143	NS

#### CPT-II performance in relation to CT, MDQ and bipolar disorder

Among the ADHD patients 59 % had a positive score on the test for cyclothymic temperament. In Table 5 are shown CPT-II data from these patients, separated into two groups based on scores on this test. For reaction time, omission and commission errors, variability and ADHD Confidence index there were no significant differences between the two groups. However, the ADHD group with cyclothymic temperament had higher values for SD (37.2 ± 12.1 vs. 26.8 ± 7.5, *p* = 0.004, *d* = 0.98) and RMSSD (45.6 ± 17.7 vs. 31.3 ± 9.4, *p* = 0.005, *d* = 0.95) and also lower values for sample entropy (1.48 ± 0.25 vs. 1.68 ± 0.20, *p* = 0.011, *d* = −0.86), while skewness was higher (3.45 ± 2.93 vs. 1.86 ± 1.29, *p* = 0.032, *d* = 0.65). For the clinical controls those with cyclothymic temperament had similar scores on the CPT-II test as those without this temperament (data not shown). For these four measures we also tested the difference between all four groups using ANOVA. This showed significant results for all measures except symbolic dynamics: F (80,3) = 12,232, *p* < 0.001 for SD; F (80,3) = 10,560, *p* < 0.001 for RMSSD; F (80,3) = 10,977, *p* < 0.001 for sample entropy and F (80,3) = 3,059, *p* < 0.033 for skewness. Post hoc Bonferroni tests showed that the ADHD + CT group was significantly different from all the three other groups for SD (*P* = 0.003, <0.001 and <0.001), RMSSD (*P* = 0.004, <0.001 and <0.001), and sample entropy (*P* = 0.027, <0.001 and <0.001). For skewness the ADHD + CT group was not significantly different from the ADHD and no CT group.

**Table 5:** ADHD patients with and without cyclothymic temperament

I Traditional CPT-II results				
	CT + (*n* = 29)	CT - (*n* = 20)	P	*d*
Reaction time	384 ± 97	383 ± 112	NS	0.01
Omissions	3.1 ± 3.2	4.0 ± 7.0	NS	−0.18
Commisions	57.0 ± 25.7	52.7 ± 22.8	NS	0.03
Variability	15.1 ± 10.2	15.0 ± 19.0	NS	0.01
ADHD Confidence Index	66.6 ± 20.9	61.0 ± 22.3	NS	0.26
II Analyses based on extraction of raw data from the CPT-II test				
	CT + (*n* = 25)	CT - (*n* = 16)	P	*d*
Reaction time	369 ± 93	393 ± 118	NS	−0.23
SD (% of mean)	37.2 ± 12.1	26.8 ± 7.5	0.004	0.98
RMSSD (% of mean)	45.6 ± 17.7	31.3 ± 9.4	0.005	0.95
Sample entropy	1.48 ± 0.25	1.68 ± 0.20	0.011	−0.86
Symbolic dynamics	74.1 ± 17.2	80.4 ± 14.9	NS	0.39
Skewness	3.45 ± 2.93	1.86 ± 1.29	0.032 ^a^	0.65

^a^ MannWhitney U-test, t-test for the other data

When the ADHD patients are separated into two groups based on the presence or not of positive score on the MDQ questionnaire there were no significant differences (data not shown). Similarly, for ADHD patients with a comorbid bipolar diagnosis, there were no significant differences (data not shown).

## Discussion

The main findings of the present study are first that adult patients with ADHD differ from clinical controls on the CPT-II test not only in increased variability, but also reduced complexity. Secondly these changes in variability and complexity are only found in the subgroup of ADHD patients that fulfill criteria for a CT.

For the whole group of patients, omission and commission errors, variability and skewness, discriminate well between patients with ADHD and those with other psychiatric diagnoses, mainly mood and anxiety disorders. The results are similar to previous studies using Conner’s CPT in adult patients. Epstein et al. [34] found more omission and commission errors in ADHD patients than in normal adults, but no difference in reaction time and variability. Similar findings concerning omission and commission errors were reported by Murphy, Barkley and Bush [35], but in addition they found higher RT variability. This was also the conclusion in a meta-analysis by Hervey, Epstein and Curry [10]. The response time distribution of ADHD patients is characterized by some very long response times and consequently high skewness, shown in studies both on children and adolescents [36–38]. This was also found in the present study, and higher skewness separated the patients with ADHD from those without this diagnosis.

We think these data support the notion that the CPT-II is a useful test in the assessment of patients with mixed complaints, including mood, anxiety and attentional problems, in an adult outpatient setting. This is in contrast to previous studies that have found little support for the usefulness of the Conner’s CPT in evaluating adult psychiatric out-patients. Walker et al. [12] found that adults with ADHD performed more poorly than normal adults, but that the test did not discriminate ADHD-patients from patients with mood and anxiety disorders. Similarly, Solanto, Etefi and Marks [13] found that the CPT-test was of little value in the differential diagnosis of adult ADHD vs. mood and anxiety disorders. Both these studies used control groups of psychiatric patients with a range of comorbid disorders, similar to what we had in our clinical control group. However, in the study of Walker et al. [12] the patients in the clinical control group were older and had higher depression scores compared to the ADHD patients, and in the study of Solanto, Etefi and Marks [13] the clinical controls were also older and a larger number had depressive disorders than in the ADHD groups. We think that these factors may have contributed to the lack of ability of the CPT to discriminate ADHD from other psychiatric disorders. Neuropsychological assessment is today not a part of the diagnostic process in adult ADHD. However, we think there is a need for more objective tests, such as CPT, in adult psychiatry, but these contrasting findings clearly show that further studies must be performed to establish the role of this test.

Assignment of an ADHD diagnosis has been based on the fulfillment of formal diagnostic criteria. Even though CPT-II has only been used as supplementary information in the diagnostic process we have used correlation between MINI+ scores and CPT-II measures to get an independent verification of the association between CPT-II and ADHD. These correlations showed the same pattern as we found when CPT-II measures were listed according to the presence or not of a final ADHD diagnosis.

In a number of studies it has been shown that increased intraindividual variability of reaction times is a characteristic feature of ADHD [1, 39, 40]. Variability, measured with the SD, is also an important parameter in a wide range of other conditions and disorders [41, 42]. Increased intraindividual variability has been found in behavioural studies with spontaneously hypertensive rats, an animal model of ADHD [43]. In agreement with this, in the present study we found an increased variability in patients with ADHD, both when assessed with variability of the standard error and with SD calculated for the whole time series. RMSSD has been less well studied as a measure of variability, but gave very similar results compared to the SD, indicating that the short term variability, from one response to the next is not substantially different from the overall variability. We have however not tried to look at variability in different frequency spectra.

Although variability has been offered much attention in the field of ADHD research, complexity has not been given a similar focus. Analysis of the complexity of the present time series show that sample entropy and symbolic dynamics gave very similar results, indicating that in adult ADHD patients there is reduced complexity of response time series, meaning increased order and predictability. This is in agreement with the suggestion that reduced complexity is associated with disease states and aging [17]. In a rat model of depression Friedman et al. [44] found reduced complexity of the firing pattern of dopaminergic neurons. On the other hand the degree of complexity may depend on the dynamics of the system that is being studied. Vaillancourt and Newell [45] suggested that systems characterized by intrinsic oscillations may show increased complexity, and this has for instance been found in a study of the motor activity of schizophrenic patients [15]. The patients we have studied all had psychiatric disorders, but problems with attention are clearly more characteristic of the ADHD patients, and we think it is meaningful that changes in variability and complexity are seen in just these patients. The present findings indicate that the study of RT variability in ADHD would benefit from including methods derived from the study of non-linear systems. This could contribute to the a better understanding of attentional fluctuations and possibly also fluctuations in mood and behaviour [46].

In neuropsychological tests, increased variability has been reported in patients with depression [42], and in studies using mood ratings over long time periods both increased variability [47] and reduced complexity [48] have been reported in patients with mood disorders. Similarly, altered variability and complexity have been found in actigraph recordings from patients with depression and bipolar disorder [19]. There is a close connection between mood disorders and ADHD, and mood symptoms in ADHD usually show a pattern with rapid and short fluctuations [5, 6]. This is reflected in the questions used in the CT questionnaire. The CT is part of the more comprehensive Temperament Evaluation of Memphis, Pisa, Paris and San Diego, auto questionnaire version (TEMPS-A), with subscales also of dysthymic, hyperthymic, irritable and anxious temperaments [8, 9]. The questions are chosen to cover behaviour and experiences reflecting traits that are characteristic of the person over time, and not only episodic manifestations as seen in depressive and manic episodes. There is substantial evidence that these affective temperaments are related to broadly defined bipolar spectrum disorders [49].

Landaas et al. [6] studied the same CT questionnaire in a sample of adult ADHD patients and found that 71 % of these patients had a cyclothymic temperament. We have extended these findings to a mixed out-patient clinical population, and find a comparable figure, 59 % prevalence of CT in adult ADHD patients. When analyzing the raw data from the CPT-II test the ADHD patients with a CT were different from those without this temperament on the two variability measures, skewness and sample entropy, even though the traditional CPT-II measures did not differentiate them. We did not find such a relationship when looking at the subgroups with comorbid bipolar disorder or positive scores on the MDQ questionnaire. This may indicate that even though episodic bipolar disorder, assessed by the MINI + or positive MDQ scores, are frequent in ADHD patients, the affective instability defined by the CT scale may reflect a more basic similarity between bipolar spectrum disorders and a large group of adult ADHD patients. The findings also attest to the usefulness of extracting raw data and performing separate analyzes on these data sets.

The present results are in apparent direct contrast with the findings of Hopwood and Morey [50], that emotional problems may suppress the relationship between continuous performance test results and ADHD symptoms. However, the designs of the studies are so different that a direct comparison is not possible.

Lundervold et al. [51] in a study on adult ADHD patients using the Attention Network Test, found that patients with affective fluctuations, defined by positive scores on the MDQ questionnaire, were more alert, but slower and more easily distracted than those without such fluctuations. Such results also attest to the importance of subdividing adult ADHD patients into subgroups based on affective symptoms.

The only difference we found between the inattentive and combined ADHD subgroups was in reaction time, while none of the other measures differed between groups. In adults Tucha et al. [52] found only small differences between ADHD subgroups using a computerized test battery measuring different aspects of attention, while Solanto et al. [13] found that the inattentive subgroup generally performed worse on the CPT-test compared to the combined subtype. We suggest that the presence of affective instability can be a more useful factor, especially in adults, to subgroup ADHD patients. This is in agreement with studies that also have separated ADHD patients according to temperament [3].

### Strengths and limitations

The patients reported on in this study may not be representative of ADHD patients in a general population. Most of them have comorbid psychiatric disorders in addition to ADHD and it is therefore difficult to know the effect of ADHD compared to the contribution from these other conditions. On the other hand, when these patients are seen in an adult psychiatric setting, comorbidity with other disorders is the rule, and also in epidemiological studies the comorbidity is high [2]. It is therefore reasonable to regard the study group as representative of adult ADHD patients coming for evaluation in a psychiatric out-patient clinic. The ADHD patients have been compared to a control group with other psychiatric disorders, but it would of course have been desirable to make comparisons also with a normal control group, matched for age and gender.

The clinical interviews were performed by two different psychiatrists, but the use of the same diagnostic instruments and a final common evaluation of each case by these two, in addition to two other experienced psychiatrists, make it likely that the diagnostic assessment is reasonably accurate.

The time interval between the presented letters (ISI) varies randomly in the CPT, and each individual therefore received a different presentation of intervals. Randomization of intervals between stimulus events has been shown to reduce variability compared to fixed intervals in a go/no-go test [53]. It is therefore conceivable that presentation rate may have influenced the non-linear measurements we have made, in particular symbolic dynamics. This should be taken into consideration when designing further studies using these methods.

We do not think medication is a likely confounding factor. Most of the patients were not using any medication, and among those that did use psychopharmacological agents, antidepressants were the most common medications, and with equal frequency in the ADHD and clinical control groups.

## Conclusion

In contrast to some previous studies, we have found that the CPT-II is a useful supplement in the diagnosis of ADHD in adult patients with a mixture of mood, anxiety and attentional problems. Furthermore, it is possible to gain additional information from this test by extracting raw data and performing separate analyzes using linear and non-linear methods. With such an approach we have found that patients with ADHD differ from clinical controls not only in showing increased variability, but also reduced complexity, and that these changes are restricted to the subgroup of ADHD patients that fulfil criteria for a cyclothymic temperament. We suggest that this may be employed not only in subtyping of ADHD patients, but also when trying to predict treatment effects.

## Abbreviations

ADHD, attention deficit hyperactivity disorder; ANCOVA, Analysis of covariance; ANOVA, Analysis of variance; ASRS, Adult ADHD Self-Report Scale; CPT, Conner’s Continuous Performance Test; CPT-II, Conner’s Continuous Performance Test II; CT, cyclothymic temperament; DSM-IV, Diagnostic and Statistical Manual of Mental Disorders, 4th Edition; HADS, Hospital Anxiety and Depression Scale; ICD-10, International Classification of mental and behavioural Disorders -10; ISI, inter stimulus interval; MADRS, Montgomery-Asberg Depression Rating Scale; MDQ, Mood Disorder Questionnaire; MINI +, Mini-International Neuropsychiatric Interview; RMSSD, root mean square successive differences; RT, reaction time; SD, standard deviation; SPSS, Statistical Package for the Social Sciences; TEMPS-A, Temperament Evaluation of the Memphis, Pisa, Paris, and San Diego autoquestionnaire; WURS-25, Wender Utah Rating Scale, 25 questions version.
